# Evidence for mTOR pathway activation in a spectrum of epilepsy-associated pathologies

**DOI:** 10.1186/2051-5960-2-71

**Published:** 2014-07-08

**Authors:** Joan Liu, Cheryl Reeves, Zuzanna Michalak, Antonietta Coppola, Beate Diehl, Sanjay M Sisodiya, Maria Thom

**Affiliations:** Departments of Neuropathology, UCL Institute of Neurology, Queen Square, London, WC1N 3BG UK; Department of Clinical and Experimental Epilepsy, UCL Institute of Neurology, Queen Square, London, WC1N 3BG UK

**Keywords:** mTOR pathway, Epilepsy, Neuronal dysplasia, Hippocampal sclerosis, Rasmussen’s’ encephalitis

## Abstract

**Introduction:**

Activation of the mTOR pathway has been linked to the cytopathology and epileptogenicity of malformations, specifically Focal Cortical Dysplasia (FCD) and Tuberous Sclerosis (TSC). Experimental and clinical trials have shown than mTOR inhibitors have anti-epileptogenic effects in TS. Dysmorphic neurones and balloon cells are hallmarks of FCDIIb and TSC, but similar cells are also occasionally observed in other acquired epileptogenic pathologies, including hippocampal sclerosis (HS) and Rasmussen’s encephalitis (RE). Our aim was to explore mTOR pathway activation in a range of epilepsy-associated pathologies and in lesion-negative cases.

**Results:**

50 epilepsy surgical pathologies were selected including HS ILAE type 1 with (5) and without dysmorphic neurones (4), FCDIIa (1), FCDIIb (5), FCDIIIa (5), FCDIIIb (3), FCDIIId (3), RE (5) and cortex adjacent to cavernoma (1). We also included pathology-negative epilepsy cases; temporal cortex (7), frontal cortex (2), paired frontal cortical samples with different ictal activity according to intracranial EEG recordings (4), cortex with acute injuries from electrode tracks (5) and additionally non-epilepsy surgical controls (3). Immunohistochemistry for phospho-S6 (pS6) ser240/244 and ser235/236 and double-labelling for Iba1, neurofilament, GFAP, GFAPdelta, doublecortin, and nestin were performed. Predominant neuronal labelling was observed with pS6 ser240/244 and glial labelling with pS6 ser235/236 in all pathology types but with evidence for co-expression in a proportion of cells in all pathologies. Intense labelling of dysmorphic neurones and balloon cells was observed in FCDIIb, but dysmorphic neurones were also labelled in RE and HS. There was no difference in pS6 labelling in paired samples according to ictal activity. Double-labelling immunofluorescent studies further demonstrated the co-localisation of pS6 with nestin, doublecortin, GFAPdelta in populations of small, immature neuroglial cells in a range of epilepsy pathologies.

**Conclusions:**

Although mTOR activation has been more studied in the FCDIIb and TSC, our observations suggest this pathway is activated in a variety of epilepsy-associated pathologies, and in varied cell types including dysmorphic neurones, microglia and immature cell types. There was no definite evidence from our studies to suggest that pS6 expression is directly related to disease activity.

## Introduction

Dysmorphic neurones (DN) and balloon cells (BC) with astroglial features are the cytological hallmarks of focal cortical dysplasia (FCD) type IIb [1]. Similar cell types are also seen in cortical lesions of tuberous sclerosis (TSC) [2]. In both pathologies, mammalian target of rapamycin (mTOR) pathway activation has been demonstrated [3–6], possibly representing a primary pathogenic mechanism and a potential target for new treatment approaches [7]. Immunohistochemical confirmation of phosphorylated downstream proteins in the mTOR pathway, particularly the ribosomal protein phospho S6 (pS6), has become a useful laboratory investigation in these pathologies. mTOR regulates many critical physiological processes governing cell survival, protein and lipid synthesis, proliferation, metabolism, autophagy and cell death in adult and developing neural tissues [8–10, 7, 4] with recent investigations demonstrating how mTOR deregulation could directly influence the cytopathology of FCD [11].

Hypertrophic neurones and astrocytes with BC-like morphology have been documented in the context of acquired epilepsy-associated pathologies, including hippocampal sclerosis (HS) [12–16] and Rasmussen’s encephalitis (RE) [17–19]. In some cases, the differential diagnosis with FCDIIb is raised by such cytopathological alterations, and the implementation of mTOR activation markers could be considered as an adjunct diagnostic test. Recent studies, however, have demonstrated mTOR pathway activation in HS in epilepsy [20, 21], suggesting that it may not be a specific biomarker for FCDIIb or TSC alone.

In this study we explored the mTOR pathway activation, through the pattern and cellular distribution of pS6 labelling, in a wide range of pathologies associated with drug-resistant epilepsy. We demonstrate immunoreactivity associated with ‘dysplasia-like’ cytopathological changes, in reactive processes, as well as in varied mature and immature neuronal and glial cell types.

## Materials and methods

### Case selection

Consent was obtained from patients for use of tissue in research and the project has ethical approval (NRES -National Research Ethics Service 12SC0669). 45 patients who had undergone epilepsy surgery were selected from the databases of the Epilepsy Society Brain and Tissue Bank at UCL Institute of Neurology. The range of pathologies studied is detailed in Table 1 with patient demographics. They included samples from 32 cases representative of lesional epileptogenic pathologies and 18 samples with no specific epileptogenic lesion; in five patients, more than one tissue sample was used representing lesional and extra-lesional tissue. Three non-epilepsy surgical control samples were included. All epilepsy patients had undergone neurosurgery for the management of drug-resistant epilepsy. The pathological criteria for HS and FCD types were based on the current International League Against Epilepsy (ILAE) classifications [22, 1].

**Table 1:** **Pathology groups and clinical data**

	Pathology group	Case	Age of onset of seizure (years)	Age at resection (years)/Gender	Outcome	Resection site/Procedure	Main pathology features in section
	N = number of cases		(Seizure type)				
**Epilepsy-lesional pathology**	HS N = 4	1	4 (PS, GS)	34 M	SF	Right ATL	HS ILAE type I: neuronal loss in CA1, CA4, gliosis, granule cell dispersion; mossy fibre sprouting confirmed in 3 cases.
		2	6 (PS, GS)	43 F	NSF	Right ATL	
		3~	1 (PS)	55 F	NSF	Left ATL	
		4	31 (PS, GS)	46 M	NSF	Left ATL	
	HS With dysmorphic neurones n = 5	5	UK	30 F	NSF	Left ATL	Hippocampal sclerosis (ILAE type I) with dysmorphic neurones in CA4 and balloon cell glia in DG: mossy fibre sprouting confirmed in 3 cases.
		6	12	42 F	NSF	Right ATL	
		7	7 (PS, GS)	31 F	SF	ATL	
		8	25 (GS)	42 F	SF	Right ATL	
		9	14 (PS, GS)	54 F	NSF	Right ATL	
	FCDIIA N = 1	10~	7 (PS, GS)	18 F	NSF	Right parietal resection	Dysmorphic neurones; no balloon cells.
	FCDIIB N = 5	11~	UK (PS, GS)	26 M	SF	Right temporal lobectomy	Dysmorphic neurones, cortical dyslamination and balloon cells
		12	11 months (PS)	24 F	SF	Left parietal resection	
		13~	7 (GS)	34 F	SF	Right frontal resection	
		14	15 months (PS)	33 F	NSF	Right parietal resection	
		15	5 (PS, GS)	18 M	NFS	Right parietal resection	
	FCD IIIA N = 5	16	12 (PS)	54 F	SF	Right ATL	Neuronal loss in outer cortical layers with gliosis and reorganisation of layer II neurones
		17	2 (PS)	34 M	SF	Right ATL	
		18	18 (PS, GS, SE)	44 M	SF	Left ATL	
		19	8 (PS, GS)	40 M	SF	Left ATL	
		20	3 (PS, GS)	19 F	SF	Left ATL	
	FCD IIIB N = 3	21	10 months (PS, GS)	27 F	SF	Left temporal lobectomy	Cortex adjacent to a long-term epilepsy associated tumour (LEAT/DNT)
		22	6 (PS, GS)	23 M	SF	Right ATL	
		23	7 (PS)	31 F	SF	Temporal lobectomy	
	FCD IIID N = 3	24	3 (PS, GS)	18 F	NSF	Right hemispherectomy	Cortical disorganisation adjacent to an early infarct
		25	4 (PS)	23 M	NSF	Left ATL	
		26	11 (PS, GS)	18 F	NSF	ATL	
	Rasmussen’s Encephalitis N = 5	27	11	18/F	NSF	Right sided brain biopsy	Active encephalitis* + gliotic atrophic cortex
		28	3 (FS, GS)	30 M	SF	Right hemispherectomy	Active encephalitis* + neuronal hypertrophy
		29	14 (FS, GS, EPC)	18 M	SF	Temporal lobe resection and hemispherectomy	Active encephalitis* + normal cortex and atrophic cortex and frequent dysmorphic neurones
		30	UK	9 F	NSF	Left temporal lobe resection	Burnt out encephalitis, atrophic cortex
		31	UK	UK	NSF	Cortical resection	Burnt out encephalitis, atrophic cortex
	Cortex adjacent to Cavernoma	32	27 (FS)	30 M	SF	Right temporal lobe resection	Cavernoma with reactive gliosis including ‘balloon cell’ like glia
**Control-non lesional**	Acute ICE injury N = 5	11~	12 (S, GS)	26 M	SF	Right temporal lobectomy	Organising electrode track cavity of 8 days
		33	15	30 F	SF	Left ATL	Organising electrode track cavity of 8 days
		10~	7 (FS, GS)	18 F	NSF	Right parietal resection	Organising electrode track cavity of 8 days
		12~	11 months (FS)	24 F	SF	Left parietal resection	Organising electrode track cavity of 10 days
		13~	7 (GS)	34 F	SF	Right frontal resection	Organising electrode track cavity of 10 days
	Epilepsy: Paired samples from different regions according to intracranial recordings. N = 4	34	6 (GS)	39M	NSF	Right frontal resection:	No specific pathology- focal inflammation
						Sample 1. Ictal onset zone	
						Sample 2. Peripheral samples in CUSA specimen	
		35	7 (PS, GS)	25 F	SF	Left frontal lobe resection	No specific pathology
						Sample 1. Seizure onset zone	
						Sample 2. Frontal pole; spreading of EEG activity	
		36	16 (PS)	33 M	SF	Right frontal lobe resection	No specific pathology
						Sample 1. Ictal onset zone	
						Sample 2. Inferior fronto-orbital; spreading of activity	
		37	8 (PS,GS)	37 M	SF	Right frontal lobe resection	Focal inflammation only
						Sample 1. Ictal onset zone	
						Sample 2. Spreading of EEG activity	
	Epilepsy: Pathology negative	38	12 (GS)	18 M	NSF	Frontal lobe cortex	Pathology negative
		39	18 (PS, GS)	31M	NSF		
		40	6 (PS, GS, SE)	34 M	SF	Temporal lobe cortex	No pathology in temporal lobe; HS in other sample
		41	6 (PS, GS)	43 F	SF		
		3~	1 (PS)	55 F	NSF		
		42	31 (PS, GS)	46 M	NSF		
		43	UK	40M	UK		
		44	6 (PS)	48 F	NSF		
		45	7 (PS, GS)	48 M	SF		
	Non-Epilepsy Cortex N = 3	46	NA	37M	NA	Left temporal lobectomy	Metastatic carcinoma
		47	NA	30M	NA	Left temporal lobectomy	Low grade oligo-astrocytoma
		48	NA	74M	NA	Right temporal lobectomy	High grade astrocytoma

ICE = Intracranial electrode, FCD = focal cortical dysplasia, HS = hippocampal sclerosis, DG = dentate gyrus, GS = generalised tonic clonic seizures, PS = partial of focal seizures (no distinction made between complex and simple type), SE = status epilepticus, EPC = Epilepsy partialis continua, SF = completely seizure free at follow up, NSF = not seizure free at follow up (includes nocturnal seizure and rare seizures/auras) [Follow up periods vary between 1 year to 15 years and status taken at last follow up], ATL = anterior temporal lobectomy including hippocampectomy, DNT = dysembryoplastic neuroepithelial tumour, ICE = intracranial electrode injury, CUSA = ultrasonic tissue aspirator, DG = dentate gyrus, UK = unknown. *Active encephalitis was determined by the presence of microglial nodules/neuronophagia and lymphocytic infiltrates on H&E as well as HLADR and CD163 labelling. ~These cases had more than one lesion in different regions of the surgical resection.

**Figure 1:**
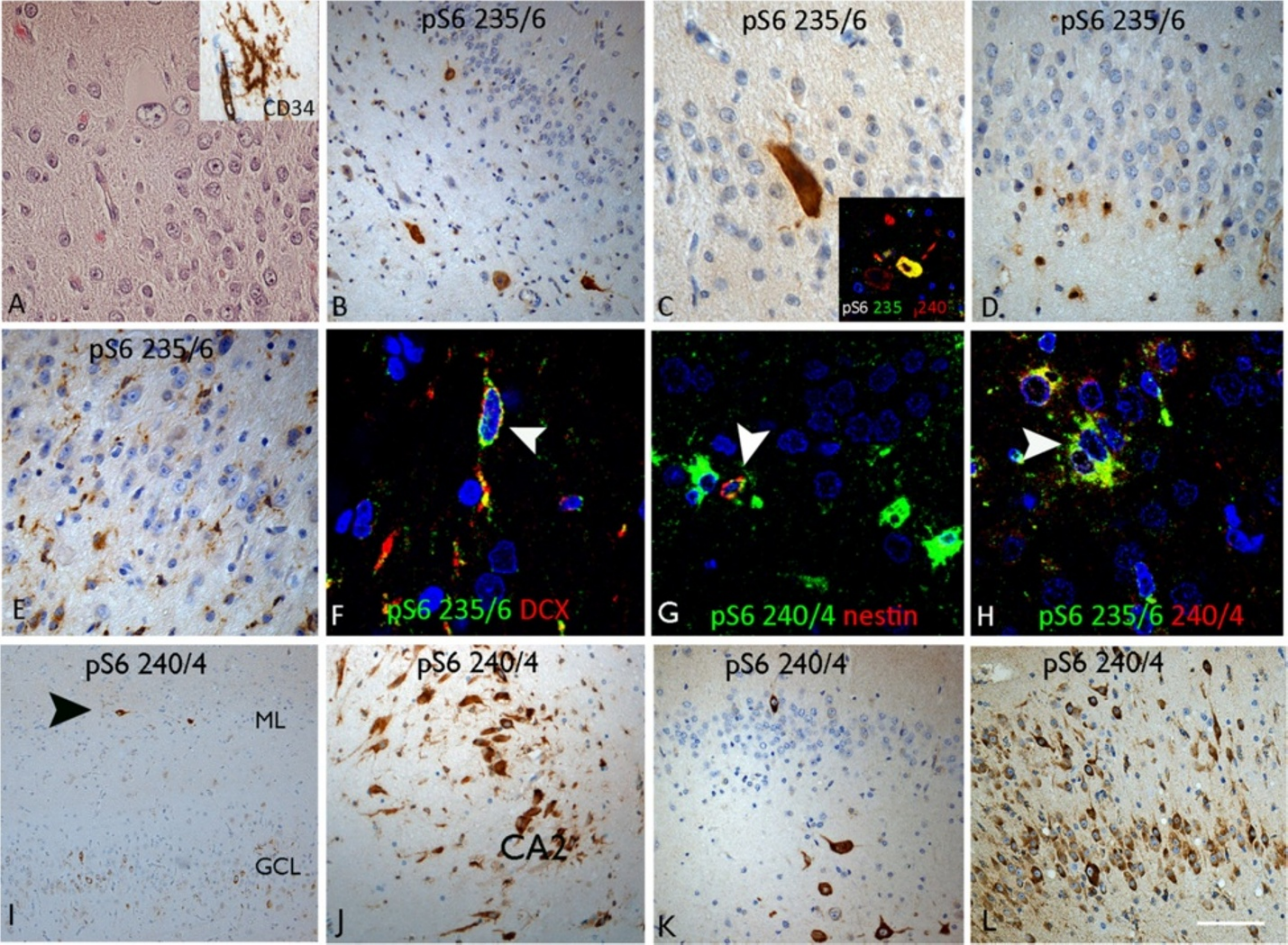
**pS6 in HS ILAE type 1 with (A-J) and without (K-L) dysmorphic changes.** HS ILAE type 1 with dysmorphic changes **(A-J) (A)** The dentate gyrus in a case with ILAE HS type 1 and additional glassy balloon-like astroglial cells on H&E which show membranous positivity for CD34 (inset). **(B)** pS6 235/236 labelling of hypertrophic CA4 neurones and **(C)** in the granule cell layer was noted in HS cases with dysplasia features; inset shows co-localisation of labelling in a proportion of cells with the two pS6 antibodies. **(D)** pS6 235/236 also labelled small immature cells with bipolar or multipolar processes including in the basal layer of the dentate gyrus **(D)** as well as through the dentate gyrus **(E)**. Co-localisation between doublecortin (DCX) and pS6 was noted in some of these small cells in the dentate gyrus **(F)** as well as with nestin **(G)**; in addition both pS6 markers co-labelled a proportion of small multipolar cells in HS. **(I)** With pS6 240/244 prominent labelling of horizontal cells in the stratum moleculare of the hippocampus, in addition to more distinct labelling of pyramidal cells through hippocampal subfields was noted. **HS ILAE type 1:** Intense labelling of CA4 neurones but the the granule cells were more variably negative **(K)** or positive **(L)** with pS6 240/244. White arrowheads in all images indicate double-labelled cells. Bar in **A**, **C**, **D**, **E**, **F**, **G**, **H** and J equivalent to approximately 35 microns and in **B**, **I**, **K** and L approximately 50 microns.

**Lesional cases** included patients with mesial temporal lobe epilepsy (mTLE) and HS ILAE type 1 with (n = 5) or without (n = 4) additional dysplasia-like cytopathological changes, including prominent dysmorphic, neurofilament-positive neuronal cells in CA4 and CD34-positive BC-like astrocytes in the dentate gyrus as previously described [15, 14, 13, 12] (Figure 1A). FCD was represented by type IIa (n = 1), type IIb (n = 5), type IIIa (dysplasia associated with HS; n = 5), type IIIb (dysplasia associated with dysembryoplastic neuroepithelial tumours (DNT)/CD34-positive long-term epilepsy-associated tumours (LEAT); n = 3), type IIId (dysplasia associated with an early infarct; n = 3) and a cavernoma with florid adjacent reactive gliosis (n = 1). In FCDIIb cases TSC was excluded clinically. We included resections from patients with a clinical and radiological diagnosis compatible with RE (n = 5) who had undergone either diagnostic biopsy or therapeutic neurosurgical procedure. The stages of inflammatory activity and scarring varied both within and between cases, as detailed in Table 1, and in two cases with RE, cortical neurones appeared hypertrophic and dysmorphic.

**Control groups with no epileptogenic lesions** included epilepsy patients who had undergone intracranial EEG monitoring where two separate samples were available from grid or electrode sites according to regional differences in ictal activity (n = 4), as detailed in Table 1. We also included a further pathology-negative group (n = 5) where only localised acute cortical injuries were identified within the tissue specimens following previous depth electrode insertion for intracranial EEG monitoring (8 to 10 days prior to tissue resection), as previously studied [23, 24]. Further control groups included surgical cortical resections from patients with epilepsy, but no cortical injuries, or lesional pathology (n = 9), and from patients without epilepsy which represented marginal normal cortex to a neoplasm (n = 3).

### Immunohistochemistry

For the demonstration of pS6, we utilised two antibodies recognising different phosphorylation sites of pS6: ser240/244 specific for mTORC1 pathway, and ser 235/236 which is a phosphorylation site that may be mTOR-independent through Ras-MAPK pathway [25]. For this purpose, 5 μm thickness formalin-fixed, paraffin-embedded brain sections of each case were processed through xylene and graded alcohols before immersion in a solution with 0.9% hydrogen peroxide for 15 minutes. Sections were microwaved in unmasking buffer (H-3301; Vector Laboratories Inc., USA) at full power for 12 minutes, and cooled for 20 minutes. Sections were blocked using 2.5% normal horse serum (Vector Lab, Peterborough, UK) for 20 minutes before incubation in a solution containing anti-phospho-S6 ser240/244 (1:1000, #5364, Cell Signaling Technology, Inc., Danvers, MA, USA) or ser235/236 (1:150, #4857, Cell Signaling Technology, Inc.) overnight at 4ºC. For RE cases, immunolabelling with HLADR (1:100, Monoclonal, Mouse HLA-DP, DQ, DR Ag, Clone: CR3/43, DAKO, Cambridgeshire, UK) and CD163 (1:2000, Monoclonal, Mouse Clone: EDHu-1, AbD Serotec, Oxfordshire, UK) was carried out to localise the regions of active chronic inflammation. The following day, DAKO REAL Envision horseradish peroxidase (HRP) solution (DAKO, Cambridgeshire, UK) was applied for 30 minutes and diaminobenzidene chromogenic activation was performed. Immunolabelled sections were counterstained with haematoxylin (VWR International, Leicestershire, UK), then coverslipped.

For double-labelled immunofluorescence, a similar protocol was applied except sections were incubated overnight at 4ºC in a primary antibody solution containing anti-phospho-S6 Ribosomal proteins (ser240/244 or ser235/236). On the following day, species-specific HRP secondary solution (Vector Laboratories Inc., Peterborough, UK) was applied for 30 minutes, before fluorescein-labelled antibody in tyramide signal amplification (TSA) buffer (1:500, Perkin Elmer, Massachusetts, UK) was applied for eight minutes. The TSA system is a sensitive detection system used in previous human tissue studies (Thom et al., [26]). Sections were thoroughly washed using phosphate buffer saline (PBS), and then immersed in 0.9% hydrogen peroxide solution for ten minutes before anti-doublecortin (DCX) (1:250, Cell Signaling Technology Inc. USA), anti-CD34 (1:25, DAKO, Cambridgeshire, UK), anti-nestin (1:1000, Abcam, Cambridge, UK), anti-Iba1 (1:1000, WAKO, Osaka, Japan), anti-SMI32 (1:1000, Sternberger Monoclonals, Baltimore, MD, USA), anti-GFAPdelta (1:4000, Abcam, Cambridge, UK) or anti-GFAP (1:100, DAKO, Cambridgeshire, UK) diluted in DAKO antibody diluent was applied overnight at 4°C. The next day, sections were washed and incubated in species-specific peroxidase solution for 30 minutes before rhodamine-labelled antibody in TSA buffer (1:500, Perkin Elmer, Massachusetts, USA) was applied for eight minutes. After PBS washes, sections were coverslipped using DAPI mounting medium (Vector Laboratories Inc., Peterborough, UK).

The cellular staining and distribution was assessed qualitatively using brightfield (Nikon Eclipse 80i), epi-fluorescence (Zeiss Axio Imager Z2), and confocal laser scanning microscopes (LSM-Meta 710, Zeiss, Germany)

## Results

### HS ILAE Type 1 with dysmorphic neurones

In these cases with histological features as previously reported [15] (Figure 1A), strong labelling of hypertrophic neurones in CA4 and dentate gyrus was observed using anti-pS6 (ser235/236; Figure 1B,C) with weak or absent labelling of other neuronal cells, particularly granule cells (Figure 1B-D). Prominent labelling of small, bipolar and multipolar cells was striking in the dentate gyrus and CA4 (Figure 1D,E) and to a lesser extent in other subfields and white matter. In one case (case 6), prominent labelling of such cells located along the basal layer of the granule cell layer was observed (Figure 1D). In addition, labelling for pS6 was also noted in large CD34-positive balloon-like cells (Figure 1A) in the dentate gyrus. Double labelling immunofluorescent studies showed the co-localisation of pS6 with DCX (Figure 1F), nestin, as well as pS6 (ser 240/244) with pS6 (ser235/236) in some of the small cells, particularly in the granule cell layer (Figure 1H).In comparison, anti-pS6 (ser240/244) showed more prominent neuronal labelling, including horizontal cells in the molecular layer (reminiscent of Cajal-Retzius cells) (Figure 1I) and pyramidal cells in CA1-3 (Figure 1J). Intense anti-pS6 (ser240/244) immunoreactivity was also noted in residual hypertrophic CA4 cells (Figure 1C, inset). Although less prominent than with anti-pS6 (ser235/236), the anti-pS6 (ser240/244) antibody also labelled small, multipolar cells in the hippocampus, and co-expression studies showed occasional co-localisation with nestin (Figure 1G) and DCX in the dentate gyrus.

### HS ILAE type 1

Scattered residual CA4 neurones were also intensely labelled with anti-pS6 (ser235/236), although labelling with this marker was more prominent in small, multipolar cells in CA4, the subgranular zone and CA1. Intense and frequent labelling of neurones from the subiculum to CA4 was more consistently seen with anti-pS6 (ser240/244) (Figure 1K). The granule cell layer was more-often immuno-negative with both pS6 antibodies (Figure 1K), although intense labelling of granule cells, including dispersed cells, was noted in some cases (Figure 1L).

### FCD II

**Figure 2:**
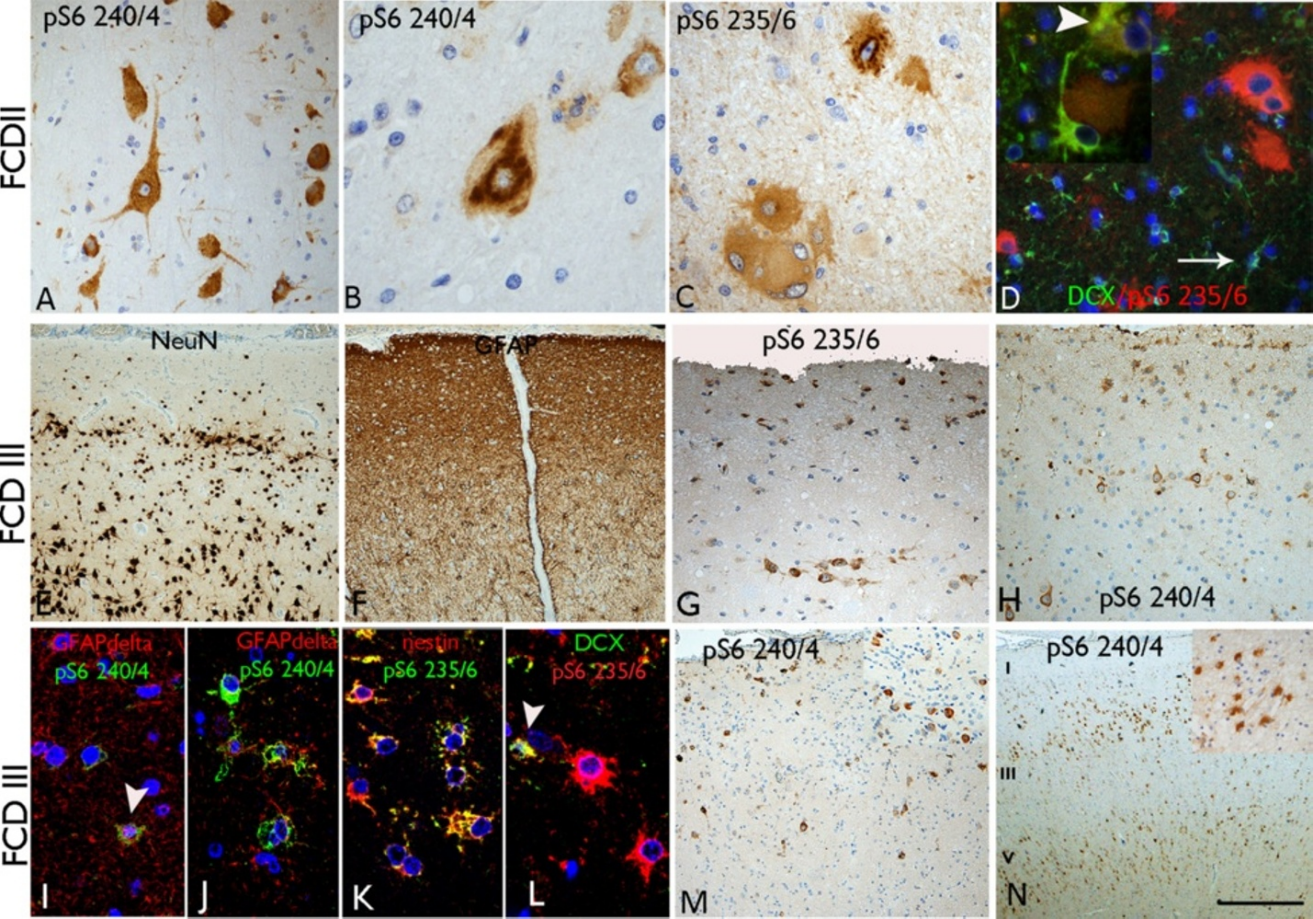
**pS6 in FCD subtypes.** FCD II: **(A)** Intense labelling was confirmed in dysmorphic neurones in FCDIIa and FCD IIb **(B)** as anticipated, highlighting the tigroid appearance of the cytoplasm **(B)**. Dysmorphic neurones were also intensely positive with pS6 235/6 **(C)**, although the cytoplasm of balloon cells appeared somewhat weaker. **(D)** Double labelling of pS6 with DCX confirmed the balloon cells as pS6 positive, with some balloon cells co-labelling with DCX (arrowhead, inset); small DCX bipolar cells were not always pS6 labelled (arrow) and wrapping of a DCX-positive cell processes around a pS6-positive balloon cell (inset) was also noted. **FCD IIIa: (E)** FCD type IIIa (adjacent to HS) with laminar neuronal loss demonstrated with NeuN labelling and clusters of neurones in layer II accompanied by mark superficial cortical gliosis **(F)**; in these cases labelling of the residual neurones in layer II with pS6 ser 235/6 **(G)** and pS6 ser 240/244 **(H)** was noted. Double labelling studies confirmed co-localisation of pS6 240/244 with GFAPdelta isoform in the cortex **(I)** and white matter **(J)** and between nestin and pS6 235/6 in small glial cells particularly in perivascular regions **(K)**, as well as focally between DCX-positive small cells and pS6 235/236 **(L). FCD IIIb:** pS6 highlighted intense labelling of scattered cortical neurones in adjacent dyslaminar cortex, as well as neurones trapped within the tumour (inset), but negative labelling of the small tumour cells **(M). FCDIIId:** Disrupted cortex adjacent to an old perinatal infarct in this case showed a prominent ‘tramline’ labelling pattern of pyramidal cells **(N)**; inset shows prominent labelling of astroglial cells in the region of chronic cortical scarring in an FCD IIId case. Bar in **A**, **B**, **C**, **D**, **I**, **J**, **K** and **L** equivalent to approximately 35 microns, in e,f,g,h and m to 50 microns and in n to 100 microns.

In the single case with FCDIIa pathology, prominent labelling of DN was seen with both pS6 antibodies, highlighting the region of dysplasia (Figure 2A), with less intense labelling of reactive glia in the region of the dysplasia; labelling of neurones in the adjacent non-dysplastic cortex was, however, also noted. In five cases of FCDIIb, DN showed a striking tigroid cytoplasmic labelling pattern with both pS6 markers, sometimes condensing in the perinuclear zone (Figure 2B,C). BC showed immunopositivity with both markers but these cells were, in general, less intensely labelled than the DN (Figure 2C). In addition, labelling of small glial-like cells (multipolar and bipolar cells) was noted in the region of dysplasia, and in morphologically normal neurones in the adjacent cortex. Double labelling with anti-pS6 (ser 235/236) and DCX showed the co-labelling of some BC, although many small DCX-positive cells were not labelled (Figure 2D).

### FCD III

These cases were characterised by neuronal loss, gliosis and associated reorganisation of cortical layers II and III with mal-orientated and clustered neurones [26, 1] (Figure 2E,F). Prominent immunoreactivity of the residual horizontal neuronal clusters in layer II for both pS6 antibodies was noted in two of five cases (Figure 2G,H). Labelling was evident in glial cells in the superficial cortex (Figure 2G) and of scattered pyramidal cells throughout the deeper cortical layers (Figure 2H) and glial cells in the white matter around vessels. Double labelling showed co-expression of pS6 markers with GFAPdelta in small cells located in the superficial cortex and in the white matter (Figure 2I,J) and with nestin, which was prominent in perivascular regions (Figure 2K). There was also evidence of co-localisation of pS6 and DCX in occasional small cells in the superficial cortex (Figure 2L). The three cases of DNT with adjacent cortex showing dyslamination at the tumour margin (**FCDIIIb**) showed strong labelling with pS6 of cortical neurones entrapped within the tumour, with tumoural oligodendrocyte-like cells strikingly immuno-negative (Figure 2M). The adjacent cortex showed labelling of glial cells in layer I and in occasional cortical neurones (Figure 2M) but the pattern and distribution, in both tumour and peri-tumoral cortex, differed to that observed with CD34 labelling. In three cases of **FCDIIId** (cortical dyslamination/disorganisation adjacent to an early infarct), intense labelling of astrocytic cells in the region of the old cortical scar was prominent (Figure 2N, inset). Labelling of scattered neurones in the adjacent cortex was seen in all cases and with a prominent ‘tramline’ pattern of labelling of pyramidal cells in layers III and V in one case (case 24) with pS6 (ser240/244) (Figure 2N). A single case of a cavernoma showed prominent pS6 labelling of reactive glia adjacent to the vascular lesion with both antibodies.

### RE

**Figure 3:**
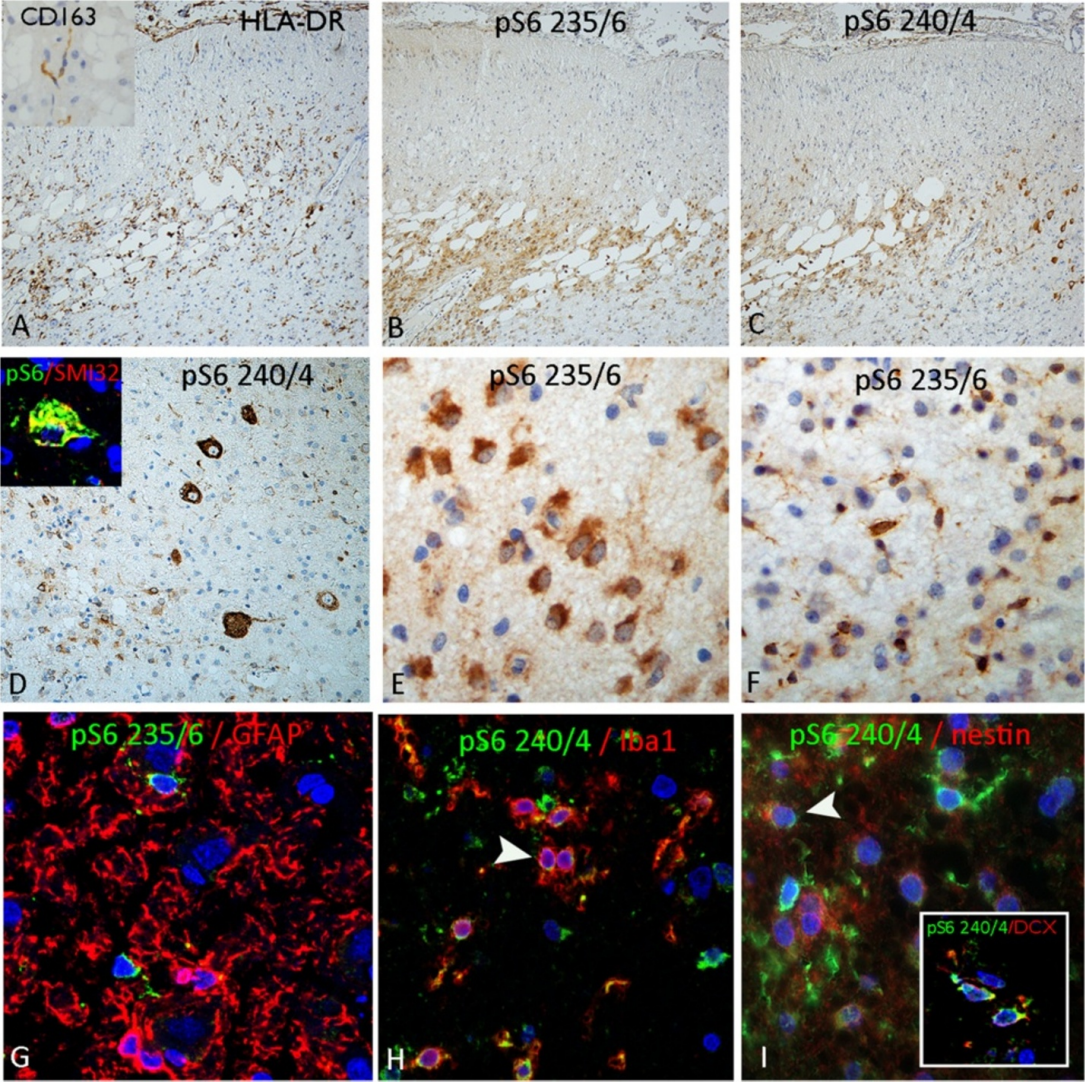
**Rasmussen’s encephalitis. (A)** Areas of active encephalitis and cortical scarring highlighted with increased numbers of HLADR-positive microglia/macrophages, as well as interstitial rod-like CD163-positive microglia cells (inset). **(B)** On adjacent sections, labelling with pS6 235/236 showed a similar distribution of cellular labelling as well as with pS6 240/244 **(C)**. **(D)** pS6 240/244 highlighted many of the enlarged dysmorphic neurones in RE cases, as confirmed with co-labelling with neurofilaments (inset). **(E)** pS6 236/26 demonstrated labelling of smaller glial cells as well as **(F)** bipolar rod shape cells. Double labelling studies in RE cases confirmed the majority of pS6 labelled cells were not GFAP-positive astroglia **(G)** but co-labelled with populations of iba1-positive cells (microglial marker) **(H)**, nestin and doublecortin (inset) **(I)** positive cells. Bar in **A**, **B**, **C** equivalent to approximately 100 microns, in d approximately 50 microns and **E**, **F**, **G**, **H**, I approximately 30 microns.

**Figure 4:**
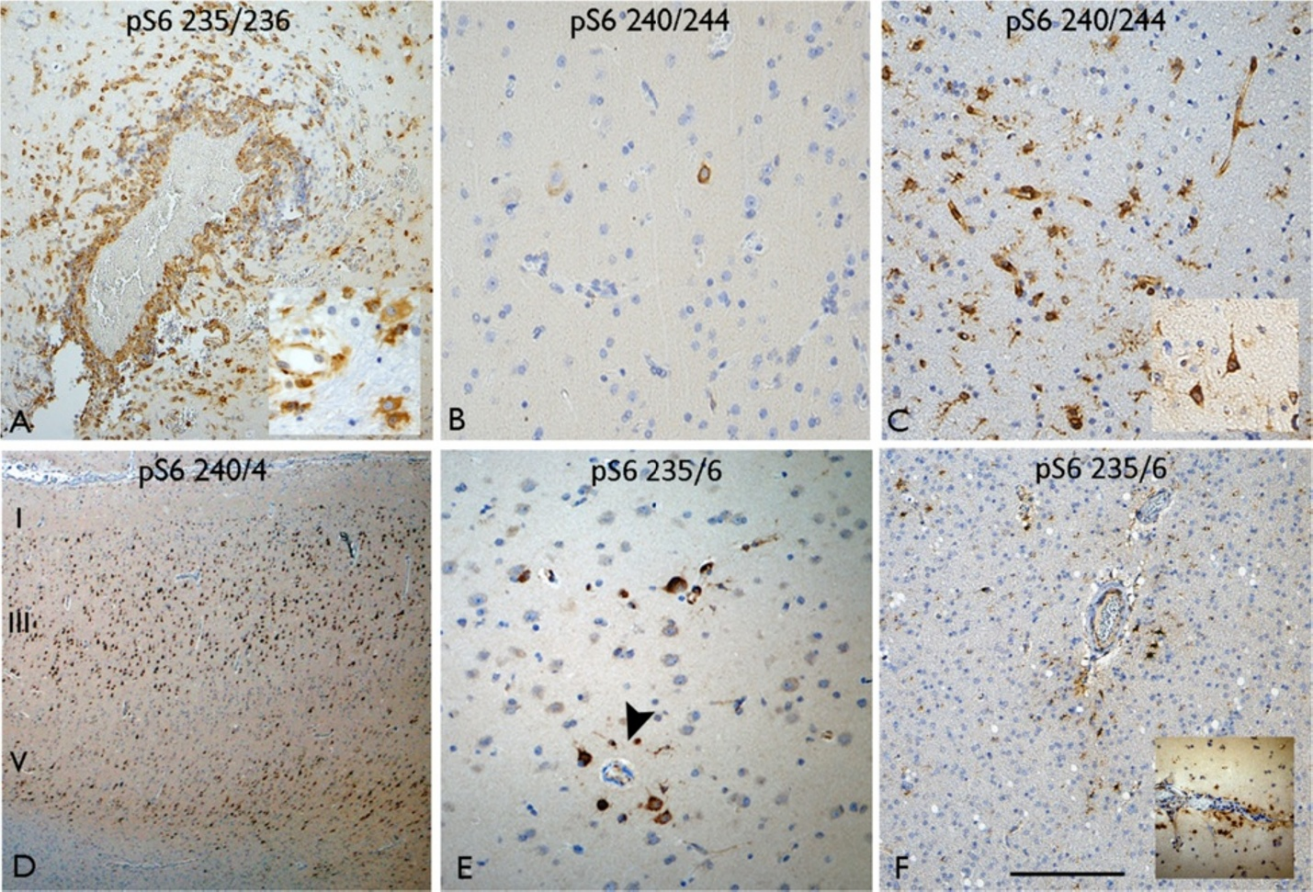
**Non-lesional epilepsy and control groups. (A)** Intense labelling of morphologically different cells types, including macrophages and multipolar cells (inset), around an organising intracranial electrode track mark. (**B** and **C**) Paired samples from regions of different ictal activity, based on intracranial monitoring: **(B)** is from case 34, sample 1 (ictal onset zone) where less pS6 labelling was seen with both markers (illustrated here with pS6 240/244) compared to **(C)** sample 2 which represented less active/seizure spreading zone where more pS6 labelling of small cells was observed as well as neuronal cells (inset). **(D)** Temporal lobe cortex with no specific pathology, adjacent to HS, where prominent ‘tramline’ labelling of cortical neurones was observed with pS6 240/244. **(E)** Another pathologically normal temporal lobe in epilepsy with a prominent perivascular labelling pattern of neuronal cells with pS6 235/236 (arrowhead) and **(F)** labelling of small glial like cells around vessels in the white matter and inset in the subpial layer (inset). Bar in **A**, **B**, **C**, **E**, **F** equivalent to approximately 50 microns and in d to 100 microns.

The stage of inflammatory activity varied between the five cases (Table 1). In two cases with extensive tissue resections, areas with active inflammation alternated with stretches of cortex showing chronic scarring and quiescent inflammation as well as regions of more normal appearing cortex; the presence of hypertrophic, dysmorphic neurofilament-positive neurones was evident in the abnormal cortex. The presence of focal, active encephalitis was confirmed with the microglial marker, HLADR (for activated microglia and macrophages), which showed increased numbers in the region of more active inflammation (Figure 3A). CD163 labelling, which shows macrophages of haemopoietic origin, also showed scattered, immunopositive, rod-like cells in these regions (Figure 3A, inset) compared to less damaged cortex, where CD163 positive cells were limited to the perivascular spaces. In the regions of active encephalitis, there was evidence of increased labelling with both pS6 antibodies (Figure 3B,C). pS6 (ser 240/244) particularly highlighted DN, whose neuronal nature was confirmed by double labelling for SMI32 (Figure 3D). pS6 (ser235/236) showed prominent labelling of small multipolar (Figure 3E) and bipolar cells (Figure 3F) in the damaged cortex; these were of similar morphology to those observed in the dentate gyrus in HS. Double labelling studies suggested the majority of pS6-positive small cells were not GFAP-expressing astrocytes (Figure 3G); but rather showed more frequent co-localisation with Iba1 (Figure 4H), nestin (in both the cortex and white matter) (Figure 4L) and to a lesser extent, with DCX (Figure 4L inset). pS6 (ser240/244) labelling of cortical neurones, including pyramidal cells in all cortical layers, was also noted in the adjacent better-preserved cortex in larger resections.

### Non-lesional cases and controls

In the five patients with acute cortical injuries following depth electrode studies, tracks lined by reactive inflammatory and granulation tissue were identified in the tissue resections. Prominent labelling with both pS6 antibodies highlighted the injury site with labelling of cell types of mixed morphologies including neurones, astrocytes, macrophages around the electrode cavity as well as vascular endothelium (Figure 4A). In addition, more widespread labelling of cortical neurones was also noted in some cases. Four patients with paired frontal tissue samples from separate sites according to intracranial electroencephalographic activities (cases 34–37, Table 1) showed no specific pathology, apart from mild inflammation. In one case (case 34), there were clear qualitative differences in the density of positive neuronal, glial and vascular labelling with both pS6 antibodies between the sample pairs (Figure 4B,C), but there were no clear differences between the samples in the three other cases. In seven non-lesional temporal lobe cortical resections adjacent to HS (cases 40–45, Table 1), scattered cortical pyramidal cells were intensely labelled with both pS6 markers. A greater proportion of neurones were positive with pS6 (ser240/244) than pS6 (ser235/236), particularly in layers III and V, imparting a ‘tramline’ labelling pattern in some cases (Figure 4D). Striking perivascular neuronal positivity was also noted in the cortex in other cases with pS6 (ser235/236) (Figure 4E). Labelling of small multipolar glia-like cells was more prominent with pS6 (ser235/236) in the white matter around vessels, in layer I and the subpial region (Figure 4F). In the pathology-negative frontal cortical resections (cases 38,39), variable patchy cortical neuronal staining of moderate intensity was also noted with pS6 (ser240/244 > ser235/236), but a tramline pattern of neuronal labelling was not apparent in frontal lobe cortex; labelling of small multipolar glial-like cells, particularly in the perivascular white matter, was also present. In the cortex from three control patients without epilepsy undergoing cortical resection for the treatment of tumours, occasional labelling for pS6 was noted in astrocytes and scattered cortical neurones.

## Discussion

Using pS6 immunoreactivity as evidence of mTOR pathway activation [27, 3, 6, 4], we have demonstrated labelling of DN in a range of mainly acquired epilepsy-associated pathologies in addition to the well-established labelling that occurs in FCDIIb [3, 6]. We have also shown pS6 expression in small, immature cell types as well as more normal neurones in epileptogenic-lesional and pathology-negative epilepsy surgical resections. The mTOR pathway is a central regulator of several vital functions relating to cell growth, proliferation and survival [8]. It was initially recognised as being pathologically activated in TSC and FCDIIb [3, 6] and subsequently in related malformative lesions primarily characterised by abnormal neuronal growth, such as hemimegalencephaly and gangliogliomas, with pS6 coming to be regarded as a biomarker of these ‘mTORopathies’ [4, 5]. Moreover, mTOR pathway activation has been reported as less evident or absent in FCD I or histologically-normal cortex in epilepsy [3, 28, 29]. We show that pS6 is not a specific marker for the cytopathology of FCD IIb or TSC in epilepsy, but more generally highlights dysmorphic cells and immature cells in epilepsy.

The mTORC1 component pathway of mTOR is the rapamycin-sensitive pathway, leading to growth-promoting conditions and increased rates of protein synthesis. The best characterized downstream targets are the eukaryotic initiation factor 4E binding protein 1 (4E-BP1) and the p70 ribosomal S6kinase 1 (S6K1) [30, 8, 4]. As pS6 is also phosphorylated by this kinase in an mTOR-independent manner [31], we used two antibodies recognising mTOR specific as well as mTOR non-specific phosphorylation sites, as employed in previous studies [6, 21], although we were unable to carry out parallel western blot analysis for cases in this study to further validate specificity. Although we noted a different range of cell expression with these two antibodies in all pathologies, co-localisation of labelling was shown in a proportion of cells. mTORC1 is normally regulated in varied physiological conditions that influence growth factors and cell energy status, through PI3K-AKT, PDK1, PTEN and other upstream signals [8, 31]. In TSC, mutations occur in *TSC1* or *TSC2* which negatively regulate mTORC1 but the mechanism of mTOR pathway activation in sporadic FCDIIb remains less well understood, with no identified pathogenic mutations. Recently Human Papilloma Virus type 16 has been detected specifically in FCDIIb as a potential acquired cause of TORC1 activation [5, 32]. Interestingly, mutations have been recently shown in an mTORC1 interacting protein, DEPDC5, in patients with malformations and epilepsy, as well as non-lesional epilepsy [33] and mTOR mutations have also been detected in epileptic encephalopathies [34] implicating this pathway may be involved in varied epilepsies.

The pathological diagnosis in FCDIIb is usually uncontroversial with standard histological stains [35]. pS6 labelling of BC with immunohistochemistry may be employed as an adjunct test, highlighting these abnormal cell populations [3, 6, 11]. This was confirmed in our series, although we noted that labelling of BC was often less pronounced than DN labelling [29]. In acquired epilepsy pathologies, dysplasia-like features may be seen, including neuronal hypertrophy with pronounced neurofilament positivity, and enlarged and hyperplastic glial cells with CD34 expression, simulating a superimposed dysplasia, which together presents a diagnostic challenge. Several reports, including cases from our own surgical series, have noted HS accompanied by hypertrophic neurones in CA4 [16, 14] or dysmorphic dentate gyrus neurones associated with prominent CD34-positive BC-like glia [12, 13, 15]. pS6 labelling in five such cases highlighted these abnormal neuronal cells in CA4 and the dentate gyrus. Previous reports of pS6 expression in dentate granule cells in HS noted labelling in a minority of cases, even in the presence of dispersion [20, 21], however when present it correlated with increased neuronal size [20]. In rodent models of HS, pS6 has been reported in the granule cells at six hours following kainate-induced seizures [20]. Interestingly, when *PTEN* was selectively deleted in granule cells (with resulting hyperactivation of mTOR), spontaneous epilepsy, granule cell hypertrophy and mossy fibre sprouting occurred [36]. In the present study, although we also noted inconsistent labelling of granule cells and other neurones with pS6 across HS cases, when present, it mainly correlated with abnormal or hypertrophic neuronal changes, which may in turn be significant to pro-epileptogenic mechanisms in addition to cytopathological alterations.

In RE, the co-existence of an FCD-like pathology is well recognised [18, 19]. In such cases, the main differential diagnosis includes FCD type II with secondary or superimposed inflammatory changes. However, the histological diagnosis of RE is made in the overall context of progressive uni-hemispheric radiological changes and lateralising neurological symptoms [37]. In cases which met criteria for RE in our series, scattered neurones with hypertrophy and dysmorphism were observed integrated within inflamed or atrophic cortex and we noted intense labelling of these DN with pS6 antibodies. Similarly, pS6 also labelled mildly enlarged, abnormally orientated layer II neurones in a proportion of FCD IIIa cases, entrapped neurones within DNT subtypes of LEAT (but not the tumoural component, as also in a recent study [27]) as well as neurones in FCDIIId. These findings all indicate pS6 as more ubiquitously present in DN in a range of epilepsy-associated pathologies. Furthermore, our studies indicate that pS6 labelling cannot reliably discriminate FCDII from RE with associated dysplasia.

Labelling neuroglial cell populations with pS6 in epilepsy pathologies, including FCD, has been recognised [29]. In HS for example, prominent and sustained labelling of astrocytes and microglia in the sclerotic hippocampus, with a relative reduction of neuronal staining was reported [21]. In that study, as little astroglial pS6 expression was identified in chronic neocortical scars, it was suggested that mTOR activation in hippocampal astrocytes was of relevance to epileptogenesis [21]. A similar study of HS in mTLE, also commented on intense labelling of astrocytes in hippocampal subfields and the dentate gyrus as well as in rodent models after 5 days of seizure induction [20]. Again, the interpretation was that over-activation of mTOR in astroglia in the more chronic phases of HS may nurture a local pro-epileptogenic microenvironment. In addition to HS cases, we noted pS6 labelling of astroglial cells in other pathologies including adjacent to electrode track injuries, one cavernoma with adjacent cellular gliosis as well as the long-standing gliosis associated with perinatal infarcts and FCDIIId. In pathology-negative epilepsy controls, only focal labelling of glial cells was seen, with minimal labelling in non-epilepsy controls. This observation of sustained pS6 labelling of astrocytes, localising to epileptogenic pathologies, would support the hypothesis of a role in promoting pro-epileptogenic glial cell activity. We also showed pS6 labelling of microglial cell types in RE and in macrophages around organising electrode tracks. mTOR, through IL-2 induction of T-cell proliferation [38], can influence inflammatory processes [39]; in turn, mTORC1 activation is regulated by inflammatory activity, for example through cytokines such as TNFα [8]. A correlation was recently shown between pS6 and inflammatory pathway activation in LEATs [27]. Our findings also highlight a potential interplay between the mTOR pathway and chronic inflammatory processes in RE which could be further investigated.

It is known that mTOR impacts on normal stem cell development and proliferation [30] and the maintenance of neural stem cells and progenitor cells [40]. We were interested therefore to explore mTOR activation in immature cell types, including in the dentate gyrus, an established neurogenic niche which may be influenced by seizures in adulthood [41]. A previous study of pS6 in HS/mTLE did not confirm pS6 in NG2-positve progenitor cells [21]. We have recently reported immature, DCX-positive bipolar cells in the dentate gyrus in HS in adults (Thom et al. 2014 In press, Psychological Medicine). In this present study we observed pS6 expression in these immature cell types, as well as in morphologically similar DCX-positive cells in RE cases. Similarly, immunostaining for nestin and GFAPdelta, developmentally regulated intermediate filaments [42], highlighted populations of immature glia in CA4 and the dentate gyrus in HS, as previously reported [43]. We have also recently identified nestin-positive glial cells in epilepsy cortical and white matter resections as representing a transient proliferative cell population, important in normal brain repair processes [24]. In this current study we also noted mTOR activation of similar immature cell types in HS as well as FCD III cases, which could highlight a functional role in the patho-aetiology of acquired epilepsy pathologies.

We aimed to address any differences in pS6 expression in direct relation to seizure activity by studying separate tissue samples with no specific pathologies but from areas of differing electrical activity based on intracranial recordings prior to the surgery. We were not able to confirm consistent differences in pS6 neuronal-glial labelling between samples. Although we did not see cavities relating to subdural grid placement or electrode track marks in these paired samples, we cannot exclude that nearby reactive inflammatory processes influence pS6 expression and mask any differences related to disease activity. Furthermore, pS6 expression was previously reported to be virtually absent in pathology-negative cortex in epilepsy [3] as well as in more recent studies [27, 44] although labelling was noted in non-sclerotic hippocampal neurones [21]. We confirm some degree of pS6 neuronal and glial labelling in all pathology-negative epilepsy cases as well as non-epilepsy controls.

There is experimental evidence implicating dysregulation of mTOR more generally in the processes of epileptogenesis, including acquired pathologies [45, 7], dysregulation being also detectable in the absence of a pathological lesion [46]. mTOR inhibitors, such as rapamycin and its analogues, have been shown experimentally to ameliorate seizures and reverse potential pro-epileptogenic cellular alterations [47–49], for example through inhibition of mossy fibre sprouting [45, 50, 51]. mTOR inhibitors have recently been approved for the treatment of some TSC lesions with success in reversal of growth of lesions [45, 7, 52, 53]. Experimental studies also show that mTOR inhibition has anti-epileptogenic effects in TSC, although the precise mechanisms are unknown [39]. Our current findings suggest that mTOR dysregulation may well be more generally implicated in pathogenesis of varied acquired lesions in focal epilepsies, particularly in the presence of dysmorphic cytopathology; the causes of this deserve further investigation. If more evidence emerges of the anti-epileptogenesis and seizure-control capacity of mTOR modulators, it may be that such therapies will find application across a broader range of epilepsies.

## Abbreviations

BC: Balloon cell
DCX: Doublecortin
DN: Dysmorphic neurone
DNT: Dysembryoplastic neuroepithelial tumour
FCD: Focal cortical dysplasia
GFAP: Glial fibrillary acidic protein
GFAPdelta: Delta isoform of GFAP
HS: Hippocampal sclerosis
ILAE: International league against epilepsy
LEAT: Long term epilepsy associated tumour
mTLE: Mesial temporal lobe epilepsy
mTOR: Mammalian target of rapamycin
pS6: Phosphorylated ribosomal S6 protein
RE: Rasmussen’s encephalitis
TSC: Tuberous sclerosis.
